# Death of Dr. John F. Brooke, U. S. N.

**Published:** 1850-02

**Authors:** 


Dr. John F. Brooke, Fleet Surgeon of the U. S. Squadron in the East Indies, died at Macao, on the 17th of October, 1849. Dr. Brooke had been twenty-five years in the service.

